# Should We Operate on Thoracic Aortic Aneurysm of 5–5.5cm in Bicuspid Aortic Valve Disease Patients?

**DOI:** 10.26502/fccm.92920230

**Published:** 2021-12-03

**Authors:** Katelyn Monaghan, Felix Orelaru, Aroma Naeem, Rana-Armaghan Ahmad, Xiaoting Wu, Karen M Kim, Shinichi Fukuhara, Himanshu J Patel, G Michael Deeb, Bo Yang

**Affiliations:** 1 Department of Cardiac Surgery, Michigan Medicine, Ann Arbor, Michigan, USA; 2 Department of General Surgery, St. Joseph Mercy, Ann Arbor, Michigan, USA

**Keywords:** Bicuspid aortic valve, Long-term outcomes, Thoracic aortic aneurysm

## Abstract

**Background:**

This study aims to determine the long-term outcomes and rate of reoperation among BAV patients with aortic diameter of 5–5.5cm who underwent immediate surgical repair versus surveillance.

**Methods:**

A total of 148 BAV patients with aortic aneurysm measuring 5–5.5cm were identified between 1993 to 2019. Patients were categorized into two groups: immediately operated (n=89), versus watched group (n=59) i.e., monitored until either symptomatic, aortic diameter ≥ 5.5 cm or operated at surgeons’ discretion/patient preference.

**Results:**

Compared to the immediately operated group the watched group had significantly lower proportion of proximal aorta replacement (86% vs 100%). The mean size of proximal thoracic aorta at initial encounter, including aortic root, ascending, and arch, for the watched group was 52.1 ± 1.62mm and 52.6 ± 1.81mm in the immediately operated group, p=0.06. There was no significant difference in 10-year survival between the watched group 94% (95% CI: 79%, 99%) vs immediately operated group 96.5% (95% CI: 86%, 99%), p=0.90. Initial operation rate for the watched group during 10-year follow-up was 85%. The operative mortality in both groups was 0%. The 10-year reoperation rate between groups was similar: 3.5% (95% CI: 0.9%, 9.1%) in the immediately operated group vs 7.7% (95% CI: 2.4%, 17.1%) in the patients who eventually had surgery in the watched group, p= 0.30.

**Conclusions:**

Our study showed that the rate of reoperation was similar between groups and survival outcomes were acceptable in observed asymptomatic BAV patients without significant family history and with proximal aortic diameter of 5–5.5cm.

## 1. Introduction

Bicuspid Aortic Valve Disease (BAV) patients with an aneurysmal proximal thoracic aorta have a higher risk of acute aortic dissection compared to TAV patients [1]. Acute type A aortic dissection is a feared complication of thoracic aortic aneurysm because it is associated with a mortality rate of 1–2% per hour after the onset of symptoms, and about 90% deaths within 90 days if left untreated [2]. Hence, the American Association for Thoracic Surgery (AATS) guidelines favor aortic repair when the proximal thoracic aortic aneurysm diameter is ≥ 5.5 cm in patients without significant risk factors [3]. However, data from the international registry of aortic dissection (IRAD) showed that majority of acute dissections occur at a proximal aortic diameter < 5.5cm [4]. There is limited evidence to guide surgeons when to operate on BAV patients with proximal aortic aneurysm. Our study aims to determine the long-term survival outcome and rate of reoperation among BAV patients with proximal thoracic aortic diameter of 5–5.5cm who underwent immediate surgical repair versus surveillance with Computed Tomography (CT) and echocardiogram between 1993–2019 at our institution.

## 2. Material and Methods

This study was approved by the Institutional Review Board at Michigan Medicine (HUM 001118517; September 26th, 2016) and a waiver of informed consent was obtained.

## 3. Data Collection

Data from 1993–2019 was retrieved and collected from the Michigan Medicine Bicuspid Aortic Valve (BAV) Registry. The BAV registry was developed to better characterize patients with bicuspid aortic valve disease by assessing patterns of aortic dilation, potential genetic markers, and the effects of medical intervention in this population. Patients 18 years and older with BAV disease were recruited for the registry. Patients with aortic aneurysms of the root, ascending, or arch measuring between 5–5.5 cm were identified by Transesophageal Echocardiogram (TEE) and Computed Tomography (CT) imaging. Data collection included pre-operative, intra-operative, and peri-operative variables as well as reoperation data. These variables were supplemented with data from the Society of Thoracic Surgery (STS) Michigan Medicine Cardiac Surgery Data Warehouse and retrospective medical record review. Survival data was obtained from the National Death Index Database through December 31st, 2018.

## 4. Patient Selection

**Figure 1:**
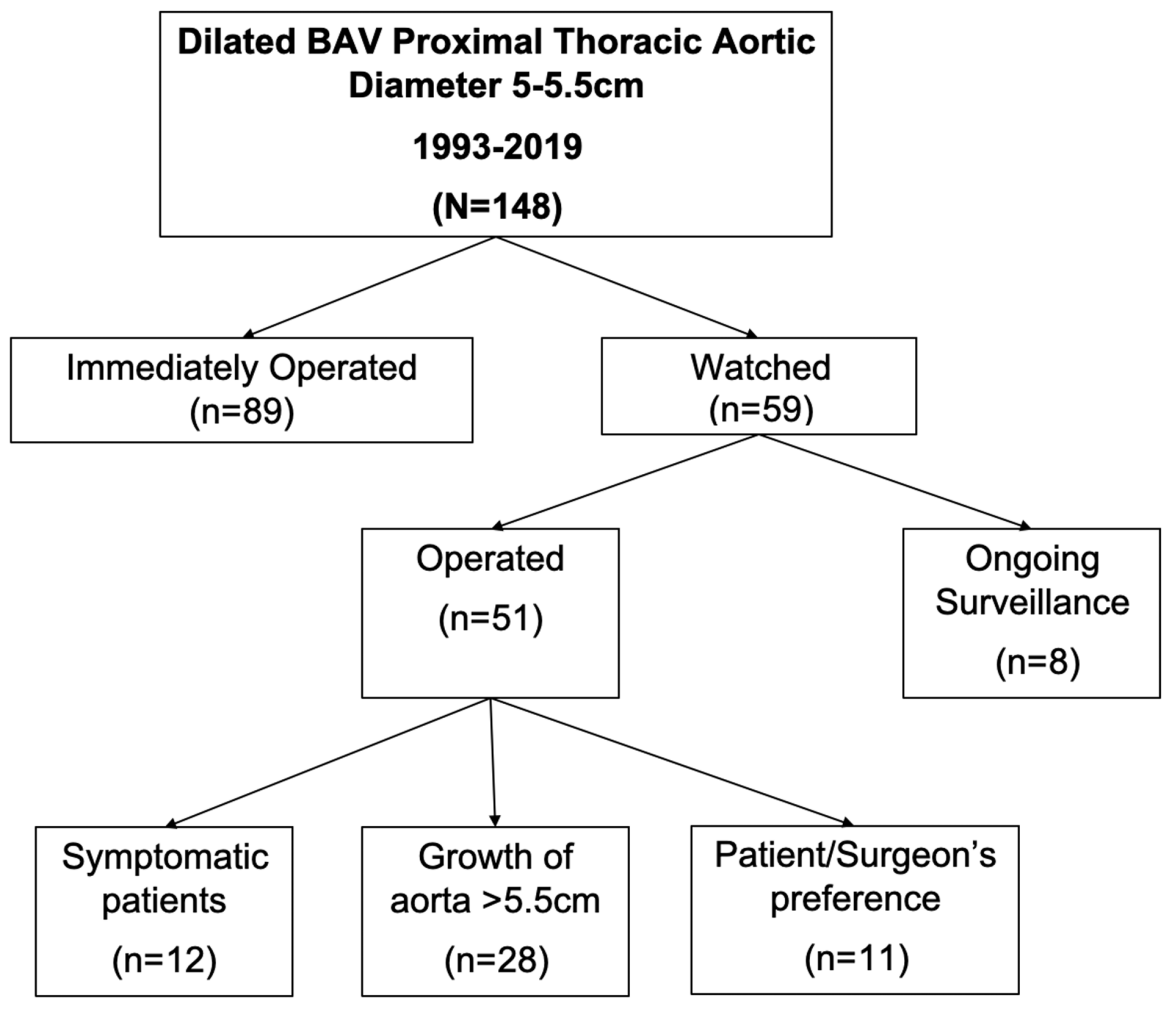
Consort diagram of selection and distribution of study population.

Between 1993–2019 a total of 148 BAV patients were identified as having aortic root, ascending aorta, or aortic arch diameters between 5–5.5 cm. Patients were organized into two groups: those who were operated on immediately for the aortic aneurysm (n=89) and those who were watched (n=59). Determination to observe or operate on patients was made at time of initial surgical consultation. Patients in the immediately operated group were scheduled for surgery at their initial consultation, whereas patients in the watched group were evaluated and followed with CT or echocardiogram imaging until either symptomatic, aortic diameter ≥ 5.5 cm, or operated on at surgeons’ discretion/patient preference. The average time between initial screening and surgery was 79 days for the operated group and 269 days for the watched group. The mean follow-up imaging time in the watched group was five years at our institution. In the watched group, 25 patients had growth of their proximal thoracic aorta diameter exceeding 5.5cm, while another 15 patients became symptomatic due to valvular dysfunction, necessitating surgery. Furthermore, there were 11 patients who underwent aortic repair due to patient preference/surgeon’s discretion. The average aortic diameter for these patients was 50.9mm. Lastly, 8 patients are still currently monitored and have not yet had an operation (Figure 1). Similarly, in the immediately operated group, 38 patients underwent aortic repair due to average aortic size of 52.2mm, 24 patients due to significant valvular dysfunction and another 27 patients were operated on due to surgeons’ discretion/patient preference (average aortic size was 52.3mm).

## 5. Statistical Analysis

Data are presented as median (interquartile range: 25%, 75%) for continuous data and n (%) for categorial data. Univariate comparisons between the watched and immediately operated groups were performed using Wilcoxon rank-sum tests for continuous data and chi-square tests for categorical data. P<0.05 was considered statistically significant. Cumulative incidence function curves using the Fine and Gray sub-distribution method with death as a competing factor were used to model the incidence of initial operations of the watched group and reoperations for both watched and operated groups over time. The Gray’s test was used to determine statistical significance between the cumulative incidence function curves of the watched and operated groups. Survival was estimated by the Kaplan-Meier method with log-rank testing. Statistical calculations were executed using SAS (SAS Institute, Cary, NC).

## 6. Results

### 6.1. Demographics and preoperative outcomes of the immediately operated group and watched group patients

Compared to the immediately operated group, the watched group had a significantly lower proportion of male patients (76% vs 94%), p=0.001 and patients with severe aortic stenosis (12% vs 33%), p=0.03. Also, patients in the watched group had a smaller body surface area (BSA) when compared to the operated group (2.1 vs 2.2), p=0.02. There was no difference between groups for all other preoperative data including hypertension, family history of aortic dissection, BAV type, aortic stenosis, or aortic insufficiency (Table 1). The initial proximal thoracic aortic mean size was similar between groups at initial consultation for surgical repair: 52.1 mm (interquartile range: 51.8–52.4) in the watched group and 52.6 mm (interquartile range: 52.2–53.1) in the operated group, p=0.06 (Table 1).

### 6.2. Intraoperative and Perioperative Outcomes

Compared to the immediately operated group, the watched group had a significantly lower proportion of proximal aorta replacement during follow-up (86% vs 100%), p=0.0004. The proportion of aortic root replacement was similar between groups (operated: 48% vs watched: 41%), p=0.52, but compared to the immediately operated group, the watched group had a significantly lower proportion of ascending aorta replacement (85% vs 94%), p= 0.05 (Table 2). Patients in the operated group had a higher incidence of perioperative atrial fibrillation compared to the watched group (38% vs 20%), p=0.04 (Table 3). Otherwise, there was no difference in intraoperative and perioperative outcomes between groups.

### 6.3. Long term Outcomes, Survival, and Reoperation

**Figure 2:**
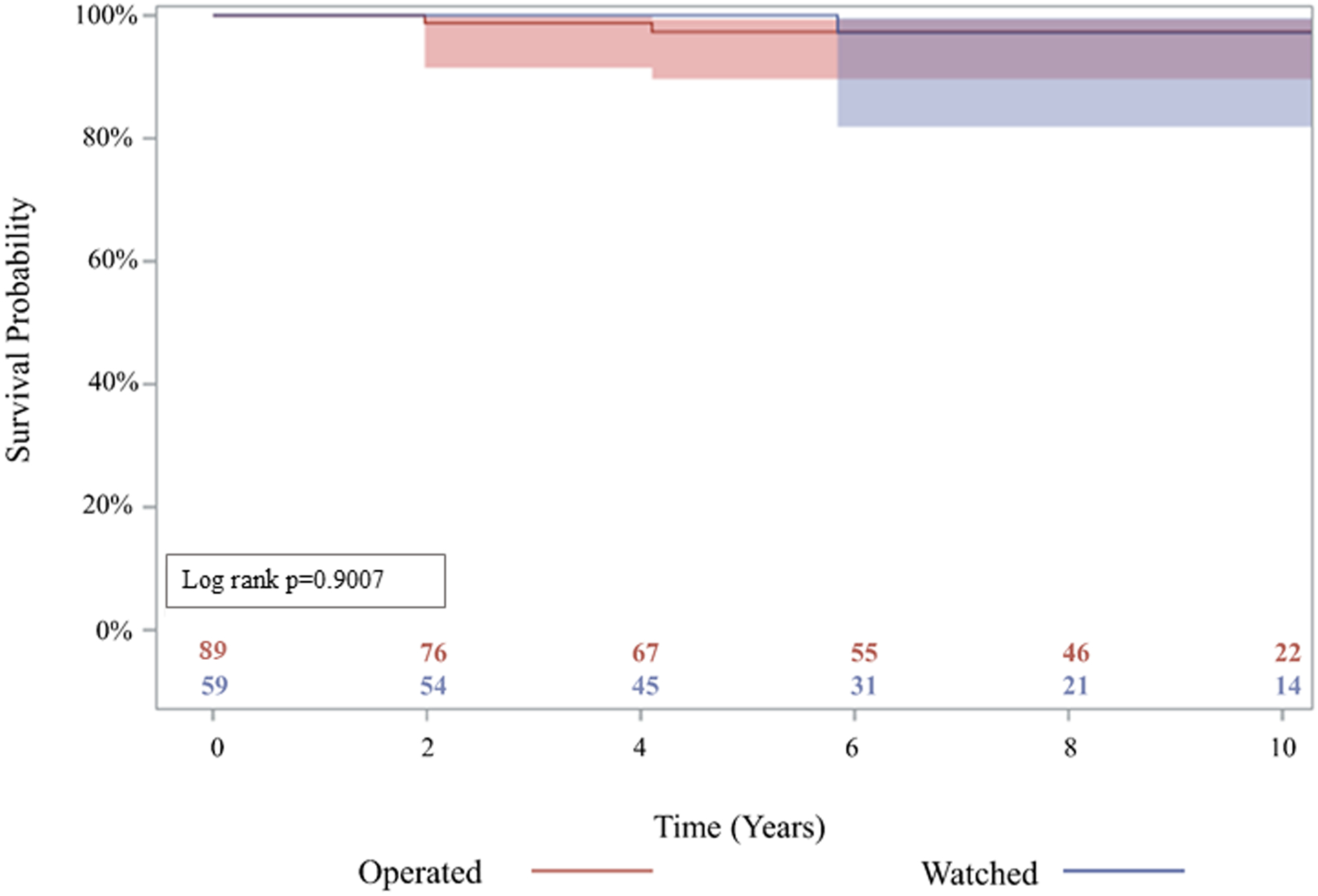
Kaplan-Meier analysis showed the long-term survival was not significantly different between operated and watched groups. 10-year survival was 96.5% (95% confidence interval (CI): 86.3, 99.1) vs 94.3% (95% CI: 79.1, 98.6), p=0.9007

**Figure 3:**
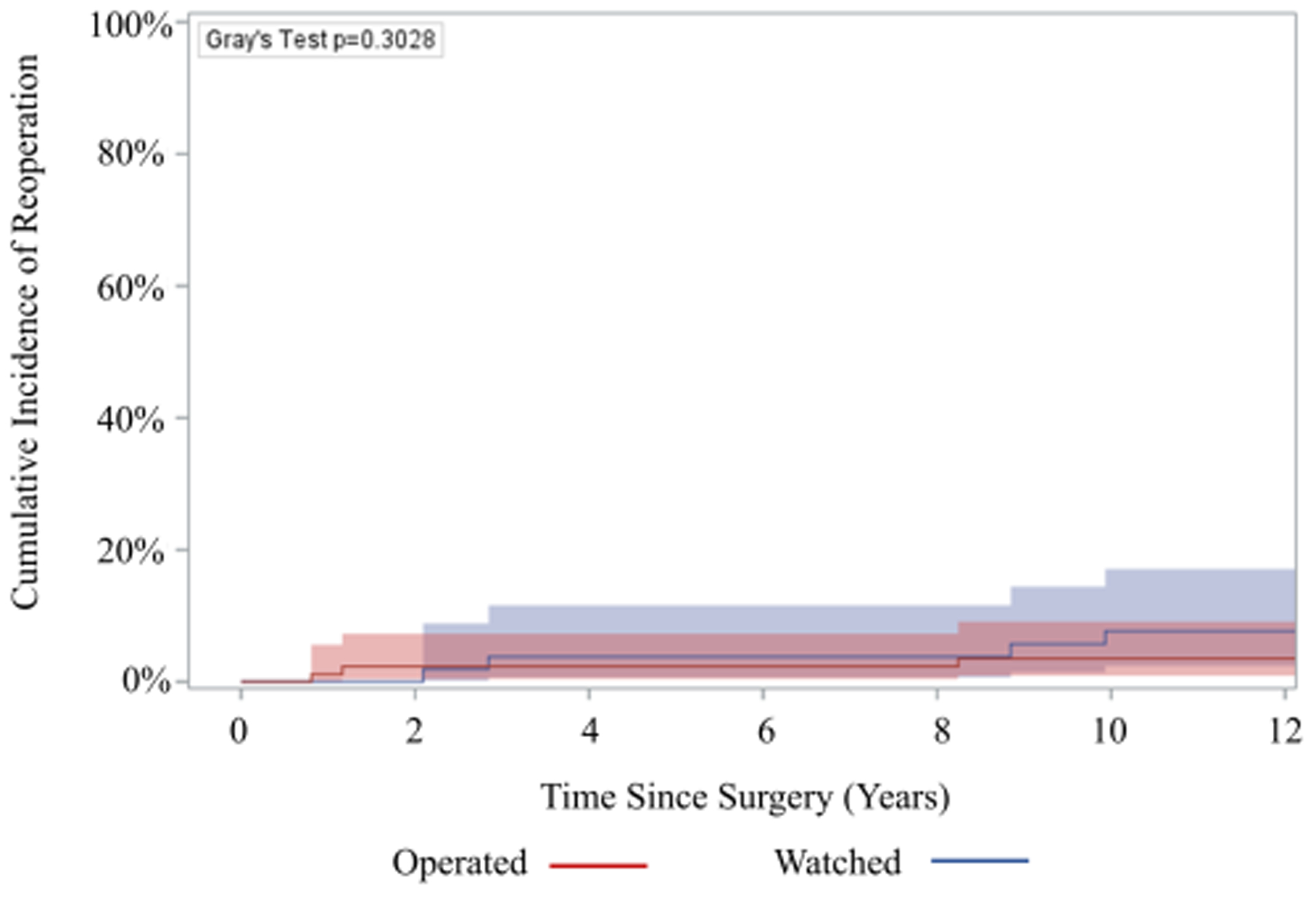
The 10-year incidence of reoperation was not significantly different between operated and watched groups: 3.5% (95% Confidence Interval (CI): 0.9%−9.1%) vs 7.7% (95% CI: 2.4%, 17.1%), p=0.3028.

There was no significant difference in late complications such as stroke (3.4% vs 0%), aortic stenosis (17% vs 20%), aortic insufficiency (37% vs 48%), or endocarditis (1.7% vs 1.1%), among others, between the watched and operated groups respectively (Table 4). 85% of patients in the watched group eventually had an initial operation during follow-up within the study period. The operative mortality was 0% in both groups (Table 3). Furthermore, 10-year survival was 94.3% (95% CI: 79.1%, 98.6%) for the watched group, and 96.5% (95% CI: 86.3%, 99.1%) for the operated group; p=0.9007 (Figure 2). There was no significant difference in 10-year reoperation rates between groups: 7.7% (95% CI: 2.4%, 17.1%) for the watched group versus 3.5% (95% CI: 0.9%, 9.1%) in the operated group, p=0.3028 (Figure 3). Indications for reoperation include aortic insufficiency, aortic root pseudoaneurysm, and endocarditis (Table 5).

## 7. Discussion

Historically, some surgeons chose a more aggressive surgical approach towards the mildly dilated proximal thoracic aorta in bicuspid aortic valve disease patients because a few genetic and observational studies showed that BAV aortopathy was associated with increased risk of acute thoracic aortic dissection compared to the general population [6,7]. This notion was underscored by data from the international registry of aortic dissection which showed that most thoracic acute dissections occur at a proximal aortic diameter < 5.5cm [4]. However, the American Association for Thoracic Surgery consensus guidelines favor aortic repair when proximal thoracic aortic aneurysm diameter is ≥ 5.5 cm in patients without significant risk factors [3]. This recommendation was based on observational studies that demonstrated an inflection point and a significant risk of aortic complications at thoracic aortic diameter of 6.0cm [8].

In our study, there was no reported incidence of acute thoracic aortic dissection in the watched group over 10 years. However, approximately 19% of patients underwent proximal aortic replacement due to patients’/surgeons’ preference, 42% of patients later had a proximal aortic diameter exceeding 5.5cm and another 25% had valvular dysfunction such as aortic insufficiency and aortic stenosis, necessitating surgical aortic repair. Fewer patients in the watched group underwent proximal aortic replacement compared to the immediately operated group (86% vs 100%) throughout the entire study period and some patients are still being observed. In line with our findings, Paruchuri et.al (2015) showed that compared to patients with Tricuspid Aortic Valve (TAV), there is no significantly increased risk of aortic dissection associated with dilated BAV aortic diameter between 5–5.5cm [8]. Also, though thoracic aortic aneurysms are more common in BAV patients compared to TAV patients, recent benchtop biomechanical data shows higher longitudinal and circumferential tensile strength and collagen stiffness in resected proximal thoracic aortas of BAV patients compared to TAV patients [9,10]. In addition, a recent study show that in an ex vivo setup, BAV proximal aortic aneurysms have greater resistance to aortic dissection compared to TAV aortic aneuryms [11].

Furthermore, our study showed that the 10-year incidence of initial operation in the watched group was 85% but the reoperation rate was low. Clearly, most BAV patients with dilated proximal aorta would require surgery when symptomatic or when aortic diameter exceeds 5.5 cm, however delaying surgery in asymptomatic BAV patients with dilated proximal thoracic aorta of 5–5.5cm is appropriate due to increased risk of surgical morbidity and mortality, especially at low volume hospital centers [12]. Also, though thoracic aorta replacement surgery is safe and favorable long term survival outcome has been documented in literature [13,14,15] perioperative outcomes such as atrial fibrillation, among others, can be mitigated by imaging surveillance of the dilated proximal aorta (5–5.5cm) in asymptomatic patients. Our study showed that there was lower incidence of atrial fibrillation in the perioperative period (20% vs 38%) in the watched grouped compared to the immediately operated group. Literature shows that postoperative atrial fibrillation is associated with prolonged hospital stay and increased healthcare costs and is an independent predictor of adverse outcomes such as kidney failure, stroke, and hemodynamic compromise after cardiac surgery including thoracic aorta replacement [16,17,18,19].

In addition to the perioperative complications, surgeons should also be aware of other long-term complications. For example, when we replace BAV patients’ proximal aorta with or without aortic valve replacement, we put them at a life-time risk of graft infection, prosthetic valve endocarditis and thromboembolic strokes. In our study, after the initial aortic aneurysm repair, both groups had these complications. Two patients in the watched group had strokes after they had aortic aneurysm repair during follow-up for proximal aortic size > 5.5cm and the other due to surgeon’s preference: the patient had moderate aortic insufficiency and stable aorta diameter size of 5.2cm. Long-term complications due to aortic grafts, valves, or anastomotic dehiscence were not high, but not zero. If patients do not need an aortic replacement, we could observe those patients to avoid the long-term complications of surgery.

Lastly, there was no survival benefit of immediate aortic repair in asymptomatic BAV patients with dilated thoracic aortic diameter between 5–5.5cm. The 10-year survival outcome in the immediately operated group was similar to the watched group (both ≥ 94%). Little evidence exists in literature about the long-term survival outcomes of asymptomatic BAV patients with dilated proximal thoracic aorta of 5–5.5cm. Masri et.al (2016) show that the 7-year survival outcome in BAV patients without surgical repair of their dilated thoracic aorta was favorable but lower than patients who underwent repair (88% vs 95%) [20]. Most patients in their study had aortic diameter less than 5.0cm and only 3.5% of patients had proximal aortic diameter between 5–5.4cm. We believe that asymptomatic BAV patients with aortic diameter between 5–5.5cm could be safely observed with favorable long term survival outcomes.

This study was a single center retrospective research study, and the sample size was relatively small. However, our study findings support the current American Association of Thoracic Surgery guidelines that favors aortic repair when the proximal thoracic aortic aneurysm diameter exceeds 5.5 cm in patients without significant risk factors. We have been increasing observation instead of operation in those patients and have been enrolling them into our BAV registry. Lastly, it is plausible that patients operated on immediately had some other consideration or risk factor not captured in our registry, that made both the surgeon and patient more inclined to opt for immediate surgery.

In conclusion, our study showed that survival outcomes were acceptable in observed asymptomatic bicuspid aortic valve patients without significant family history and with proximal aortic diameter of 5–5.5cm. While further studies are needed to reinforce this guideline, our study supported that these patients can be safely monitored with routine imaging studies until they became symptomatic or aortic diameter exceeds 5.5 cm. Though patients could be operated upon if risk of monitoring outweighs benefits based on surgeons’ clinical judgement.

## Figures and Tables

**Table 1:** Demographics and Preoperative Variables.

	Operated (n=89)	Watched (n=59)	p-value
Age	57 (50, 64)	54 (49, 62)	0.19
Sex (Male)	84 (94)	45 (76)	**0.001**
BSA	2.2 (2.1, 2.3)	2.1 (2.0, 2.2)	**0.02**
Hypertension	38 (43)	27 (46)	0.53
Diabetes	8 (9.0)	4 (6.8)	0.57
Smoking status			0.89
Never	46 (52)	30 (51)	0.92
Former	33 (37)	21 (36)	0.85
Current	6 (6.7)	6 (10)	0.54
Unknown	4 (4.5)	2 (3.4)	1.0
Dialysis	0 (0)	0 (0)	1.0
Previous MI	9 (10)	2 (3.4)	0.20
CVA	3 (3.4)	2 (3.4)	1.0
PVD	3 (3.4)	2 (3.4)	1.0
Liver disease	2 (2.3)	1 (1.7)	1.0
Chronic lung disease	6 (6.7)	9 (15)	0.09
Mild	4 (4.5)	7 (8.5)	0.48
Moderate	0 (0)	2 (3.4)	0.16
Severe	2 (2.3)	0 (0)	0.52
BAV Type			0.19
0	5 (5.6)	7 (12)	0.22
1	60 (67)	38 (64)	0.74
2	10 (11)	5 (8.4)	0.62
Prior AVR	4 (4.5)	5 (8.5)	0.32
Not reported	10 (11)	4 (6.8)	0.36
Family history of aortic dissection	1 (1.1)	3 (5.1)	0.30
Cusp Calcification	37 (42)	17 (29)	0.11
Cusp Thickening	31 (35)	12 (20)	0.06
Bovine Arch	13 (15)	4 (6.8)	0.10
Aortic Stenosis	45 (51)	33 (42)	0.09
Mild	18 (40)	16 (48)	0.46
Moderate	12 (27)	13 (39)	0.23
Severe	15 (33)	4 (12)	**0.03**
Aortic Insufficiency			0.21
None	29 (33)	19 (32)	0.96
Trace	13 (15)	3 (5.1)	0.07
Mild	19 (21)	18 (31)	0.21
Moderate	20 (22)	17 (29)	0.38
Severe	8 (9.0)	2 (3.4)	0.32
Proximal aorta size*	52.6 (1.81)	52.1 (1.62)	0.06
**Preoperative complications**			
Endocarditis	1 (1.1)	0 (0)	0.41
Type B Dissection	1 (1.1)	0 (0)	1.0

Data presented as median (25%, 75%) for continuous data and proportion (%) for categorical data. P-value <0.05 indicates statistically significant difference between operated and watched groups.

* Proximal aorta size presented as mean and standard deviation.

Abbreviations: BSA=body surface area

**Table 2:** Intraoperative Outcomes.

Variable	Operated (n=89)	Watched (n=59)	p-value
**Reason for operation**			
Patients/Surgeon’s preference	27 (30)	11 (19)	0.26
Extensive aorta growth	38 (43)	25 (42)	0.47
Valvular dysfunction	24 (27)	15 (25)	0.76
Incidence			0.24
First operation	81 (94)	45 (76)	0.21
First Redo	4 (4.7)	6 (10)	0.10
Status			0.19
Urgent	5 (6.1)	1 (1.7)	0.41
Elective	80 (93)	50 (85)	0.08
Proximal aorta replacement	89 (100)	51 (86)	**0.0004**
Root Replacement	41 (48)	24 (41)	
Ascending Replacement	84 (94)	50 (85)	
AVR	27 (31)	19 (32)	0.81
Arch Replacement			0.43
None	48 (41)	32 (54)	
Hemiarch	33 (37)	21 (36)	
Zone 1 Arch	3 (3.4)	3 (5.1)	
Zone 2 Arch	3 (3.4)	2 (3.4)	
Zone 3 Arch	2 (2.3)	1 (1.7)	
Cross clamp time (minutes)	141 (110, 172)	132 (102, 171)	0.81
CPB time (minutes)	179 (146, 221)	181 (156, 204)	0.62
Intraoperative blood transfusion	30 (35)	23 (43)	0.37
PRBCs (units)	0.0 (0.0. 1.0)	0.0 (0.0, 1.0)	0.90

Data presented as median (25%, 75%) for continuous data and proportion (%) for categorical data. P-value <0.05 indicates statistically significant difference between operated and watched groups.

Abbreviations: AVR = aortic valve replacement

**Table 3:** Perioperative Outcomes.

Variables	Operated (n=89)	Watched (n=59)	p-value
Hours intubated	5.1 (3.8, 9.0)	6.1 (3.4, 12)	0.48
ICU Stay (hours)	47 (27, 68)	47 (28, 71)	0.90
Blood transfusion	11 (14)	5 (8.5)	0.46
New onset renal failure	4 (4.7)	1 (1.7)	0.65
Requiring dialysis	1 (1.2)	0 (0)	1.0
Atrial Fibrillation	33 (38)	11 (20)	**0.04**
Reoperation for bleeding	1 (1.2)	1 (1.7)	1.0
Deep sternal wound infection	2 (2.3)	0 (0)	0.52
Operative Mortality	0 (0)	0 (0)	1.0

Data presented as median (25%, 75%) for continuous data and proportion (%) for categorical data. P-value <0.05 indicates statistically significant difference between operated and watched groups.

Abbreviations: BSA=body surface area

**Table 4:** Long-term outcomes.

Variables	Operated (n=89)	Watched (n=59)	p-value
Endocarditis	1 (1.1)	1 (1.7)	1.0
Stroke	0 (0)	2 (3.4) *	0.16
Valve Dehiscence	1 (1.1)	0 (0)	1.0
Graft Infection	2 (2.3)	1 (1.7)	1.0
Pseudoaneurysm	1 (1.1)	1 (1.7)	1.0
Aortic Insufficiency	44 (48)	22 (37)	0.18
Trace/minimal	31 (35)	16 (27)	
Mild	9 (10)	3 (5.1)	
Moderate	3 (3.4)	1 (1.7)	
Severe	1 (1.1)	2 (3.4)	
Aortic Stenosis	18 (20)	10 (17)	0.66
Contained Bentall Anastomotic Rupture	1 (1.1)	0 (0)	1.0

Data presented as median (25%, 75%) for continuous data and proportion (%) for categorical data. P-value <0.05 indicates statistically significant difference between operated and watched groups.

Abbreviations: BSA=body surface area

*: Strokes in two patients after they were operated.

**Table 5:** Indications for reoperation.

Variables	Operated (n=89)	Watched (n=59)	p-value
Aortic Insufficiency	2 (2.2)	3 (5.1)	0.39
Aortic root pseudoaneurysm	0 (0)	1 (1.7)	0.40
Endocarditis	2 (2.2)	0 (0)	0.52

## Abbreviations:

BAV: Bicuspid aortic valve
CI: Confidence Interval
CT: Computed tomography
IRAD: International registry of aortic dissection
TAV: Tricuspid aortic Valve
TAA: Thoracic ascending aneurysm
TEE: Transesophageal echocardiogram
